# The link between fear about COVID-19 and insomnia: mediated by economic concerns and psychological distress, moderated by mindfulness

**DOI:** 10.1017/jmo.2021.3

**Published:** 2021-01-28

**Authors:** Dirk De Clercq, Inam Ul Haq, Muhammad Umer Azeem, Samia Khalid

**Affiliations:** 1 Goodman School of Business, Brock University, St. Catharines, Ontario L2S 3A1, Canada; 2 School of Business, Monash University Malaysia, Selangor Darul Ehsan, Malaysia; 3 School of Business and Economics, University of Management and Technology, Lahore, Pakistan; 4 Riphah Institute of Clinical and Professional Psychology, Riphah International University, Lahore, Pakistan

**Keywords:** Conservation of resources theory, COVID-19, insomnia, mindfulness, strain, terror management theory

## Abstract

This paper adds to extant research by examining the relationship between employees’ fear of coronavirus disease 2019 and their suffering from insomnia. It specifically proposes mediating roles of employees' economic concerns and psychological distress and a moderating role of mindfulness in this process. The research hypotheses are tested with survey data collected through two studies among Pakistani-based professionals: 316 in study 1 and 421 in study 2. The results pinpoint a salient risk for employees who experience fear during a pandemic crisis, in that the associated economic and psychological hardships make the situation worse by undermining their sleep quality, which eventually could diminish the quality of their lives even further. It also reveals how organizations can mitigate this risk if employees can leverage pertinent personal resources, such as mindfulness.

## Introduction

Enjoying a good night's sleep, without encountering difficulty falling asleep or waking up during the night, may seem like a personal issue, but it also represents a critical concern for organizations (Barnes, Miller, & Bostock, 2017; Grunberg, Moore, & Greenberg, 2006; Pieters & Matheus, 2020). An exhausted labor force, suffering persistent sleep deprivation, creates a range of organizational risks, including unsafe practices but also a lack of energy available to devote to effective leadership or job tasks, which can compromise a firm's competitive positioning and strengths (Kucharczyk, Morgan, & Hall, 2012; Scott & Judge, 2006). Exhausted workers also might be more prone to negative work- and non-work-related attitudes, behaviors, and states (e.g., Barnes, 2012; Barnes, Miller, & Bostock, 2017; Crain, Brossoit, & Fisher, 2018; Schneider, Fulda, & Schulz, 2004). Restless nights and the resulting fatigue are especially prevalent when organizations and their work forces confront significant pressures, within or outside the workplace (Haar & Roche, 2013; Toker, Laurence, & Fried, 2015). Organization scholars and practitioners thus need insights into why some employees might be more likely than others to suffer from insomnia, as well as suggestions for tactics to limit the detrimental effects (Crain, Brossoit, & Fisher, 2018; Garcia, Bordia, Restubog, & Caines, 2018; Ng & Feldman, 2013; Ragins, Ehrhardt, Lyness, Murphy, & Capman, 2017).

Extant research into the determinants of sleep deprivation often cites individual factors such as age (Coombe, Epps, Lee, Chen, Imes, & Chasens, 2019), work involvement (Haar & Roche, 2013), and time spent at work or with family (Barnes, Wagner, & Ghumman, 2012), as well as organizational factors such as contract breaches (Garcia et al., 2018) or organizational changes (Rafferty & Jimmieson, 2017). Another pertinent *non-work*-related source of insomnia, with great relevance today, may be employees' fears about life-threatening viruses (Ahorsu, Lin, Imani, Saffari, Griffiths, & Pakpour, 2020). Prior research indicates that employees' fear of terrorism can spill over into diminished sleep quality (Toker, Laurence, & Fried, 2015); we consider whether their fear of coronavirus disease 2019 (COVID-19) similarly might increase the chances that employees suffer from insomnia, and we seek to identify pertinent factors that might explain or influence this link. In turn, we provide organizations with pertinent insights into *why* and *when* pandemic-related fears are associated with sleep problems, as well as how they can help employees deal with this challenge.

First, we propose that a critical factor that connects employees' fear of COVID-19 with their risk of insomnia is that this fear may impose significant strain on their personal lives, in both economic and psychological terms. Two theories underlie this argument: conservation of resources (COR) theory and terror management theory (Greenberg & Kosloff, 2008). According to COR theory, employees' material and mental well-being are pertinent resources that they value and seek to protect in the presence of adverse situations (Hobfoll, 2001). Terror management theory instead predicts that life-threatening events spur people's mortality salience, or the realization that death is an unavoidable part of life (Burke, Martens, & Faucher, 2010). The prospect of dying one day then serves as an important source of fear that informs how people experience their daily lives (Burke, Martens, & Faucher, 2010; Kosloff, Greenberg, Sullivan, & Weise, 2010). In line with both theories, we propose that employees' fear about COVID-19 relates positively to their insomnia, through experienced economic and psychological hardships. As prior studies show, employees' *job-*related strain is a mechanism that connects their exposure to external threats (e.g., terrorism) with detrimental performance outcomes (De Clercq, Haq, & Azeem, 2017). We add to this conversation by investigating the mediating role of the strain that employees experience in their personal realms, in response to a different external threat, namely, the COVID-19 pandemic. This strain may emerge in the form of economic worries (Hayes, VanBrackle, & Sigman-Grant, 2016) or psychological distress (Slade, Grove, & Burgess, 2011).

Second, we propose that employees' attention–awareness mindfulness is a personal resource that might serve as a *buffer* against the depletion of their resource reservoirs in response to their fear of COVID-19 (Hülsheger, Alberts, Feinholdt, & Lang, 2013; Roche & Haar, 2013), which then may help them maintain their sleep quality. Mindfulness is a broad concept that entails various sub-dimensions – such as acting with awareness, describing, observing, non-judging of inner experience, or non-reactivity to inner experience (Baer, Smith, Hopkins, Krietemeyer, & Toney, 2006) – but we focus on attention–awareness mindfulness specifically. This conceptualization captures employees' ‘enhanced attention to and awareness of [their] current experience or present reality’ (Brown & Ryan, 2003: 822), such that ‘awareness involves experiencing and perceiving reality, [and] attention guides awareness to specific elements of the experienced reality’ (Leroy, Anseel, Dimitrova, & Sels, 2013: 238). We adopt this conceptualization in light of the argument that the attention–awareness component of mindfulness – which we label as mindfulness herein, for parsimony – offers valuable and significant protection against difficult situations (Martin, Blair, Clark, Rock, & Hunter, 2018; Weintraub, Pattusamy, & Dust, 2019). Our argument for the buffering role of mindfulness also is consistent with COR theory, which predicts that the extent to which employees suffer from resource-draining situations is contingent on their access to this specific personal resource (Montani, Ilaria, Valentina, Courcy, & Gabriele, 2020; Qian, Yuan, Lim, Niu, & Liu, 2020).

### Contributions

In testing these predictions, we seek to make several contributions to extant research. First, we theorize and empirically show that employees' fear of COVID-19 associates positively with their insomnia, as explained by their economic and psychological hardships. Fears about the coronavirus may prevent employees from getting a healthy amount of sleep, due to the worries about their economic situation (Hilton & Devall, 1997) or psychological distress (Lin, Ma, Wang, & Wang, 2015) that it prompts. With this perspective, we point to the risk of a possible downward *spiral* for employees: The hardships that arise from their fear of COVID-19 may drain their personal resource bases so much that they lose sleep, which could leave them even more sensitive to the threats of the pandemic.

Second, we address calls to apply contingency approaches to organizational studies of the negative consequences of life-threatening situations (De Clercq, Haq, & Azeem, 2017; Raja, Azeem, Haq, & Naseer, 2020; Toker, Laurence, & Fried, 2015), by focusing on the pertinent role of attention–awareness mindfulness. Mindfulness serves a protective function in mitigating the work-related challenges that come with quantitative and emotional job demands (Haun, Nübold, & Bauer, 2018) or polychronicity (Weintraub, Pattusamy, & Dust, 2019). We propose that it also might protect employees who are fearful about COVID-19 from experiencing excessive economic and psychological hardships and thus sleep deprivation. This novel insight implies that the likelihood of persistent sleep deprivation, associated with fears about a global virus, might be contained by this personal feature. By examining this influence, we show that the discretionary energy employees derive from their mindfulness (Shao & Skarlicki, 2009) may be leveraged to help them cope with COVID-19 and thus contain the aforementioned downward spiral.

Third, research on the impact of COVID-19 on employees is just beginning, highlighting the need for careful assessments of how employees actually are suffering due to the pandemic. We thus test out two measures of fear of COVID-19, namely, an adapted version of an established fear of terror scale that has been used in previous organizational research (Haq, De Clercq, & Azeem, 2019), and another scale introduced recently in non-business research into mental health (Ahorsu et al., 2020). The first scale, as originally applied in the context of terrorism, gauges people's ‘frequent ruminations about future terrorist attacks, the sense that nothing can be done to avoid such attacks, the belief that terrorism will only get worse as time passes, or a feeling of a general lack of control in protecting oneself and loved ones from violence’ (Haq, De Clercq, & Azeem, 2019: 469). We adjust it to the pandemic context to gauge employees' fear of COVID-19. The second scale offers ‘a brief and valid instrument to capture an individual's fear of COVID-19,’ which ‘is directly associated with [the] transmission rate and medium (rapidly and invisibly) as well as its morbidity and mortality’ (Ahorsu et al., 2020: 2).

**Figure 1.**
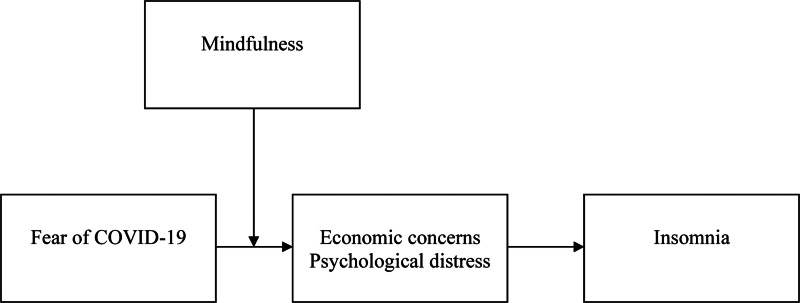
Conceptual model.

In the proposed conceptual model in Figure 1, we predict that employees' fear of COVID-19 relates positively to their economic concerns and psychological distress, which link to their suffering from insomnia. Their mindfulness buffers this process. The constitutive hypotheses – mediation by economic and psychological hardships and moderation by mindfulness – are tested in a two-study design. Study 1 investigates the mediating role of economic concerns in the relationship between fear of COVID-19 and insomnia, using the aforementioned, adapted fear of terror scale (Haq, De Clercq, & Azeem, 2019). Study 2 addresses the mediating role of psychological distress and the moderating role of mindfulness in this process, based on the scale developed for the specific case of COVID-19 (Ahorsu et al., 2020). In line with suggestions in other research into life-threatening events (Raja et al., 2020), this two-study design helps diminish response fatigue, and it provides an effective empirical test of the link between fear of COVID-19 and insomnia in two different samples. It also enables us to check whether the link is robust to the application of two different measures of fear of COVID-19.

## Study 1

### Hypotheses

A global, life-threatening virus such as COVID-19 likely has widespread economic consequences too, such as when employees suffer salary cuts or lose their jobs (Cheng, Cui, & Li, 2020; Wu, 2020). We accordingly predict that a critical factor that links employees' fear of COVID-19 with diminished sleep quality is that they worry about their economic situation. For example, an inability to keep COVID-19 out of their thoughts might make it difficult to fall asleep at night, because they doubt their abilities to provide for the basic, medical, or educational needs of themselves and their loved ones (Hayes, VanBrackle, & Sigman-Grant, 2016). This argument is consistent with a COR logic, which acknowledges that people's material needs are critical resources that inform their quality of life (Hobfoll, 2001). Their fears about the implications of COVID-19, whether actual or exaggerated, may be so upsetting for employees that they lose confidence in their ability to provide for their family or make financial ends meet, fears that keep them awake at night (Greenberg, 2006). Conversely, when employees feel less threatened by COVID-19, it is less likely that they worry excessively about their economic situation, which then has positive consequences for their sleep quality.

Hypothesis 1: Employees' economic concerns mediate the relationship between their fear of COVID-19 and their suffering from insomnia.

### Data collection

This hypothesis was tested with an online survey administered among Pakistani-based employees. The names of the employees came from a database maintained by the university of one of the authors. This database includes the contact information of employees who work for organizations that span broad sectors of the Pakistani economy. The survey was administered in April 2020 – a period that marks a high point of the COVID-19 pandemic, such that many people were limited in their physical movements and work options. The survey was administered in English, which is the formal language of business communication in Pakistan. An opening statement promised the participants complete confidentiality, clarified that no individual data would ever be released, and highlighted that the research focus was on identifying general patterns across aggregate data. This statement also specified that participants could withdraw from the study at any point in time, that there were no good or bad answers, and that the survey results would generate a better understanding of the challenges that employees encounter during the pandemic crisis. A total of 500 employees were randomly selected from the database; 316 completed surveys were returned. To ensure that the final sample included business professionals, the respondents confirmed at the start of the survey that they had been fully employed at the beginning of 2020. This sample included 71% women, 85% were 30 years or younger, 24% were married, 61% had a university degree, 35% were still earning income at the time of the data collection, and 80% worked in the private sector and 20% in the public sector.

### Measures

The constructs were measured with five-point Likert anchors, ranging between 1 (‘strongly disagree’) and 5 (‘strongly agree’).

#### Fear of COVID-19

To assess the extent to which employees are fearful about COVID-19, we adapted a 13-item measure of fear of terror (De Clercq, Haq, & Azeem, 2020b; Haq, De Clercq, & Azeem, 2019) to the context of the coronavirus. The items were preceded with a statement that asked participants to reflect on how they have been feeling about the pandemic since its outbreak. Three example items are ‘I frequently find myself preoccupied with thinking about COVID-19,’ ‘I worry that COVID-19 will only get worse as time passes,’ and ‘I think that I am completely helpless in protecting myself from COVID-19 in the future’ (Cronbach's α = .90).

#### Economic concerns

We assessed employees' worries about their economic situation with a 12-item scale of family economic strain (Hilton & Devall, 1997).^1^ This measure reflects ‘the presence of economic worry and concern about family financial problems,’ such that ‘higher scores indicate greater perceived economic strain’ (Hilton & Devall, 1997: 174). The questions were preceded by a statement that prompted respondents to indicate how they have been feeling about their economic situation in the past month. Participants then indicated their agreement with statements such as, ‘I worry about financial matters,’ ‘It is or will be hard for me and my family to live on our present income,’ and ‘Financial problems interfere or will interfere with my relationships with other people’ (Cronbach's α = .94).

#### Insomnia

We captured the extent to which employees suffer from persistent sleep deprivation with a four-item scale of insomnia (Cole, Cai, Martin, Findling, Youngstrom, & Garber, 2011).^2^ The prompt asked respondents to reflect on their sleep quality in the past few days. Two sample items are ‘It takes me a long time to fall asleep’ and ‘I wake up in the middle of the night’ (Cronbach's α = .70).

#### Control variables

The statistical models controlled for six variables: gender (1 = female), age (1 = 20 years or younger, 2 = 21–30 years, 3 = 31–45 years, 4 = more than 45 years), marital status (1 = married), education level (1 = university degree), whether the respondents were earning money at the time of data collection (1 = yes), and type of organization (1 = private, 0 = public).

### Construct validity

A three-factor measurement model, estimated through confirmatory analysis, generated an acceptable fit: χ^2^(321) = 1,159.56, confirmatory fit index (CFI) = .83, incremental fit index (IFI) = .83, Tucker−Lewis index (TLI) = .82, root mean-squared error of approximation (RMSEA) = .09, and standardized root mean square residual (SRMR) = .08.^3^ Each measurement item showed strongly significant factor loadings on its respective construct (*p* < .001; Gerbing & Anderson, 1988), which indicates convergent validity. Evidence for discriminant validity was confirmed in the significantly worse fit of the constrained models (in which the correlation between two constructs was set to equal 1), relative to the fit of their unconstrained counterparts (in which the correlation between the constructs was free to vary), for each of the three construct pairs (Δχ^2^_(1)_ > 3.84, Anderson & Gerbing, 1988).

## Results

Table 1 shows the correlations and descriptive statistics for the study variables. To assess the presence of mediation, we used the process macro (model 4, Hayes, 2013) to estimate the indirect relationship between fear of COVID-19 and insomnia through economic concerns, as well as to establish the corresponding confidence interval (CI). The findings in Table 2 indicate an effect size of .131 for this indirect relationship; the CI does not include 0 (.025, .228), in support of hypothesis 1. Fear of COVID-19 is positively related to economic concerns (β = .771, *p* < .001), which in turn associates positively with insomnia (β = .170, *p* < .01).

**Table 1.** Correlation table and descriptive statistics (study 1)

	1	2	3	4	5	6	7	8	9
1. Fear of COVID-19									
2. Economic concerns	.609**								
3. Insomnia	.402**	.336**							
4. Gender (1 = female)	.043	−.014	.112*						
5. Age	.031	.154**	−.090	−.258**					
6. Married (1 = yes)	.055	.076	−.070	−.227**	.559**				
7. Education (1 = university)	−.059	−.012	−.066	−.017	.213**	−.119*			
8. Earn money (1 = yes)	.005	−.071	−.063	−.315**	.201**	.217**	.106		
9. Organization (1 = private)	−.015	.025	−.025	−.127*	−.012	−.024	−.068	.077	
Mean	3.134	3.197	3.362	.706	1.870	.237	.611	.348	.804
Standard deviation	.738	.936	.932	.456	.703	.426	.488	.477	.398

*Note*: *N* = 316.

*p < .05; **p < .01.

**Table 2.** Mediation results (study 1)

	Economic concerns		Insomnia	
Gender (1 = female)	−.072		.140	
Age	.226**		−.097	
Married (1 = yes)	−.076		−.106	
Education (1 = university)	−.005		−.071	
Earn money (1 = yes)	−.226		.001	
Organization (1 = private)	.093		−.048	
Fear of COVID-19	.771***		.376***	
Economic concerns			.170**	
*R*^2^	.403		.198	
	Effect size	Bootstrap SE	LLCI	ULCI
Indirect effect	.131	.052	.025	.228

*Note*: *n* = 316; SE = standard error; LLCI = lower limit confidence interval; UCLI = upper limit confidence interval.

* *p* < .05; ** *p* < .01; *** *p* < .001.

## Study 2

### Hypotheses

We also hypothesize a mediating role of employees' psychological distress in the relationship between their fear of COVID-19 and insomnia. In contrast with economic concerns, this mediator reflects their experience of diminished mental health, which may manifest in a sense of nervousness, hopelessness, or depression (Bordia, Hobman, Jones, Gallois, & Callan, 2004; Van De Voorde & Beijer, 2015). According to terror management theory, fears about life-threatening events make people more aware of their own mortality, and this feeling undermines their psychological well-being (Burke, Martens, & Faucher, 2010; Greenberg & Kosloff, 2008). Furthermore, COR theory notes that adverse life situations deplete the reservoir of positive energy resources that people have at their disposal to maintain their peace of mind (Toker, Laurence, & Fried, 2015). Employees who are fearful about COVID-19 and its consequences may become so psychologically distressed that they have a hard time to falling or staying asleep (Garcia et al., 2018). Self-defeating thoughts about a dark, looming future also may evoke doubts about their worth as a person, which may be associated with diminished psychological well-being and thus a higher likelihood of insomnia (Howie, 2007; Ren, Gao, & Chen, 2020). Conversely, employees who experience low levels of psychological distress, linked to limited fears of the pandemic, should feel more energized in their daily lives and enjoy better sleep quality at night.

Hypothesis 2: Employees' psychological distress mediates the relationship between their fear of COVID-19 and their suffering from insomnia.

Consistent with COR theory, employees' psychological suffering from resource-draining life situations depends on the *severity* of the experienced resource drainage, such that their suffering is mitigated to the extent that employees can draw from valuable personal resources (Hobfoll & Shirom, 2000). We specifically propose a mitigating role of attention–awareness mindfulness (Brown & Ryan, 2003). Extant research reveals that such mindfulness enables people to separate their ‘selves’ from adverse situations and reevaluate the situations in less harmful terms (Bishop et al., 2014; Shapiro, Carlson, Astin, & Freedman, 2006). In particular, when they are equipped with attention–awareness mindfulness, people can avoid automatic negative reactions to challenging circumstances and instead experience these circumstances as less upsetting for their personal well-being (Garland, Farb, Goldin, & Fredrickson, 2015; Haun, Nübold, & Bauer, 2018). They similarly have a lower inclination to see themselves as helpless victims of experienced calamities, so they suffer less from such hardships (Glomb, Duffy, Bono, & Yang, 2011). In the terminology of COR theory or terror management theory, such processes make it less likely that employees experience a depletion of their individual resource reservoirs (Hobfoll, Halbesleben, Neveu, & Westman, 2018) or mortality salience (Burke, Martens, & Faucher, 2010), respectively.

In our study context, mindful employees may be better equipped to deal with resource-draining fears about a life-threatening virus, because their focused attention to their present reality helps them display understanding and acceptance of the pandemic situation (Chiesa & Serretti, 2009; Hyland, Lee, & Mills, 2015; Jimenez, Niles, & Park, 2010). Haun, Nübold, and Bauer (2018: 390) also indicate that awareness–attention mindfulness increases the chances that employees apply ‘an emotion-focused cognitive coping strategy through which events are re-perceived as beneficial or meaningful,’ which can diminish the probability that employees experience psychological distress, even if they are fearful about COVID-19. That is, they likely can adjust better to life-threatening events, such as COVID-19, by making sense of and deriving meaning from them (Zivnuska, Kacmar, Ferguson, & Carlson, 2016). For example, perhaps they regard the associated challenges as opportunities to achieve personal growth or display resilience (Brendel, Hankerson, Byun, & Cunningham, 2016; Evans, Baer, & Segerstrom, 2009).

Yet another argument for the buffering role of attention–awareness mindfulness is that mindful employees are more likely to realize that they are not the only ones suffering and that others likely are in similar or even more precarious situations, which tends to diminish the experience of psychological strain (Hülsheger et al., 2013; Shao & Skarlicki, 2009). Similarly, mindful employees tend to focus less on their own hardships and direct their energy toward finding ways to help other people struggling with difficult situations (Brown & Ryan, 2003; Petchsawang & McLean, 2017). Employees then may experience less psychological distress associated with COVID-19, because they can find distraction from adding to the well-being of others. In contrast, employees who are less mindful likely become more psychologically distressed by their fears about a pandemic, due to the sense that they are alone in their suffering.

These arguments, considered in tandem with the aforementioned mediating role of psychological distress, indicate a possible moderated mediation dynamic (Preacher, Rucker, & Hayes, 2007). That is, the personal resource of mindfulness serves as a critical contingency factor in the indirect relationship between employees' fear of COVID-19 and their suffering from insomnia, through psychological distress. Their mindfulness alleviates the psychological hardships associated with a life-threatening pandemic (Hülsheger et al., 2013), which then is associated with a lower probability that they suffer from persistent sleep deprivation.

Hypothesis 3: The indirect relationship between employees' fear of COVID-19 and insomnia, through psychological distress, is moderated by their mindfulness, such that this indirect relationship is weaker at higher levels of mindfulness.

### Data collection

The data collection procedure of study 2 mirrors the one used in study 1. Participants were selected from a different database of Pakistani-based employees, maintained by one of the authors. The online survey was administered in May 2020, while a national lockdown was still in place. Consistent with study 1, the opening statement that accompanied the survey guaranteed complete confidentiality and protection of participants' rights. Among the 500 people randomly selected from the database, 421 returned the survey. The respondents confirmed that they had been fully employed at the start of 2020. The final sample included 74% women, 89% were 30 years or younger, 19% were married, 71% had a university degree, 37% were still earning money at the time of data collection, and 84% worked in the private sector and 16% in the public sector.

### Measures

#### Fear of COVID-19

To assess the extent to which employees experience fear related to the coronavirus, we applied the recently developed seven-item fear of COVID-19 scale (Ahorsu et al., 2020), using five-point Likert anchors (1 = ‘strongly disagree,’ 5 = ‘strongly agree’). An accompanying statement prompted respondents to indicate their agreement with statements about how they had been feeling about COVID-19 since its inception. For example, they indicated whether ‘It makes me uncomfortable to think about COVID-19,’ ‘My hands become clammy when I think about COVID-19,’ and ‘I am afraid of losing my life because of COVID-19’ (Cronbach's α = .87).

#### Psychological distress

To measure the extent to which employees experience psychological hardships, we relied on a 10-item scale of psychological distress (Slade, Grove, & Burgess, 2011). The items asked respondents how they had been feeling over the past 30 days, such as ‘During the last 30 days, about how often did you feel hopeless/so sad that nothing could cheer you up/depressed?’ The responses were five-point Likert anchors (1 = ‘never,’ 5 = ‘all the time’; Cronbach's α = .91).

#### Insomnia

We applied the same scale to measure insomnia as used in study 1 (Cole et al., 2011). The Cronbach's alpha value of the four-item scale equaled .71.

#### Mindfulness

We measured employees' mindfulness with the 15-item mindful attention and awareness scale, developed and validated by Brown and Ryan (2003) and applied in several investigations of employee well-being (e.g., Haun, Nübold, & Bauer, 2018; Hülsheger et al., 2013; Weintraub, Pattusamy, & Dust, 2019). The items captured participants' everyday experiences; they were instructed to rate how frequently they tend to have the experiences, on six-point, reverse-coded Likert anchors (1 = ‘almost never,’ 6 = ‘almost always’). The instructions underscored that they should rate the statements according to their actual experiences, instead of what they thought their experiences should be. Three sample items were, ‘I find it difficult to stay focused on what's happening in the present,’ ‘It seems I am ‘running on automatic’, without much awareness of what I'm doing,’ and ‘I find myself doing things without paying attention’ (Cronbach's α = .88).

#### Control variables

The analyses included the same control variables as study 1: gender (1 = female), age (1 = 20 years or younger, 2 = 21–30 years, 3 = 31–45 years, 4 = more than 45 years), marital status (1 = married), education level (1 = university degree), whether the respondents were earning money at the time of data collection (1 = yes), and type of organization (1 = private, 0 = public).

#### Construct validity

A four-factor measurement model exhibited an acceptable fit: χ^2^(588) = 1,323.77, CFI = .88, IFI = .89, TLI = .88, RMSEA = .06, and SRMR = .05.^4^ All items had significant factor loadings on their respective constructs (*p* < .001), affirming the model's convergent validity. The fit of six models with constrained construct pairs was significantly worse than that of their unconstrained equivalents, which indicates the presence of discriminant validity.

### Results

Table 3 reports the correlations and descriptive statistics, and Table 4 contains the results of the mediation analysis. Similar to study 1, this analysis relied on the process macro (model 4, Hayes, 2013). We found an effect size of .095 for the indirect relationship between fear of COVID-19 and insomnia through psychological distress; the corresponding CI did not include 0 (.053, .145), in support of Hypothesis 2. Fear of COVID-19 is associated positively with psychological distress (β = .295, *p* < .001), and psychological distress in turn connects positively with insomnia (β = .322, *p* < .001).

**Table 3.** Correlation table and descriptive statistics (study 2)

	1	2	3	4	5	6	7	8	9	10
1. Fear of COVID-19										
2. Psychological distress	.459**									
3. Insomnia	.303**	.522**								
4. Mindfulness	−.369**	−.616**	−.534**							
5. Gender (1 = female)	.047	.059	.102*	−.016						
6. Age	−.026	−.153**	−.020	.103*	−.130**					
7. Married (1 = yes)	.027	−.128**	−.074	.095	−.168**	.434**				
8. Education (1 = university)	−.002	−.071	−.062	.024	.132**	.267**	.127**			
9. Earn money (1 = yes)	−.033	−.031	−.027	.041	−.112*	.081	.178**	.107*		
10 Organization (1 = private)	.002	.071	−.027	−.013	.285**	−.151**	−.215**	−.057	−.251**	
Mean	3.108	2.590	2.910	3.612	.743	1.917	.185	.713	.371	.843
Standard deviation	.816	.879	.946	.992	.437	.669	.389	.453	.484	.364

*Note*: *N* = 421.

*p < .05; **p < .01.

**Table 4.** Mediation results (study 2)

	Psychological distress		Insomnia	
Gender (1 = female)	.036		.255**	
Age	−.073		.141*	
Married (1 = yes)	−.115		−.088	
Education (1 = university)	−.077		−.156+	
Earn money (1 = yes)	.053		−.007	
Organization (1 = private)	.109		−.220*	
Fear of COVID-19	.295***		.044	
Mindfulness	−.447***		−.323***	
Psychological distress			.322***	
*R*^2^	.457		.369	
	Effect size	Bootstrap SE	LLCI	ULCI
Indirect effect	.095	.023	.053	.145

*Note*: *n* = 421; SE = standard error; LLCI = lower limit confidence interval; UCLI = upper limit confidence interval.

* *p* < .05; ** *p* < .01; *** *p* < .001.

Consistent with our conceptual arguments, the results in Table 5 indicate a negative, significant role of the fear of COVID-19 × mindfulness interaction term (β = −.103, *p* < .01) in explaining psychological distress. Similarly, diminishing effect sizes arise in the relationship between fear of COVID-19 and psychological distress at increasing levels of mindfulness (.406 at one standard deviation [SD] below the mean, .313 at the mean, .190 at one SD above the mean). To check formally for the presence of moderated mediation, we assessed the CIs for the conditional *indirect* relationship between fear of COVID-19 and insomnia at different values of mindfulness. In line with the proposed conceptual framework, the estimated model included a moderating effect of mindfulness in the relationship between fear of COVID-19 and psychological distress but not between psychological distress and insomnia (i.e., process, model 7).^5^ The results revealed diminishing effect sizes at higher levels of the moderator: from .131 at one SD below the mean, to .101 at the mean, to .061 at one SD above the mean. A formal test for the presence of moderated mediation showed that the index of moderated mediation equaled −.033; notably, its corresponding CI did *not* include 0 (−.062, −.010), in support of Hypothesis 3 (Hayes, 2015).

**Table 5.** Moderated mediation results (study 2)

	Psychological distress		Insomnia	
Gender (1 = female)	.035		.255**	
Age	−.072		.141*	
Married (1 = yes)	−.117		−.088	
Education (1 = university)	−.074		−.156+	
Earn money (1 = yes)	.042		−.007	
Organization (1 = private)	.118		−.220*	
Fear of COVID-19	.677***		.044	
Mindfulness	−.121		−.323***	
Fear of COVID-19 × Mindfulness	−.103**			
Psychological distress			.322***	
*R*^2^	.467		.369	
Conditional *direct* relationship between fear of COVID-19 and psychological distress				
	Effect size	Bootstrap SE	LLCI	ULCI
−1 SD	.406	.059	.291	.521
Mean	.313	.043	.230	.397
+1SD	.190	.057	.078	.302
Conditional *indirect* relationship between fear of COVID-19 and insomnia				
	Effect size	Bootstrap SE	LLCI	ULCI
−1 SD	.131	.031	.072	.195
Mean	.101	.024	.057	.150
+1SD	.061	.021	.022	.106
Index of moderated mediation	−.033	.013	−.062	−.010

*Notes*: *n* = 421; SD = standard deviation; SE = standard error; LLCI = lower limit confidence interval; UCLI = upper limit confidence interval.

^+^*p* < .10; * *p* < .05; ** *p* < .01; *** *p* < .001.

## Discussion

This study is timely for organizational scholars and professionals in its investigation of the possible detrimental relationship between employees' pandemic fears and insomnia, with a particular focus on key factors that underpin or influence this relationship. Despite some recent attention to how organizations and their members have reacted to the COVID-19 pandemic (Bergstrom, 2020; Snell, 2020; Vaziri, Casper, Wayne, & Matthews, 2020), this study represents a first attempt to explicate how and why a fear of COVID-19 might be negatively associated with employees' sleep quality, as well as personal conditions in which this harmful process is more or less likely to take form. The focus on insomnia as a specific outcome of pandemic fears is highly relevant, in light of the broad acknowledgment in organization research that persistent sleep failures can generate severe detrimental outcomes for employees, in both work and non-work realms (Barnes, 2012; Barnes, Miller, & Bostock, 2017; Crain, Brossoit, & Fisher, 2018). We have drawn from both COR and terror management theories to propose that (1) resource-depleting fear of COVID-19 associates positively with insomnia through employees' economic concerns and psychological distress, and (2) their attention–awareness mindfulness mitigates this process. These theoretical predictions are confirmed by the empirical findings.

The positive link between employees' fear of COVID-19 and insomnia, through their worries about their economic situation and their tarnished psychological well-being, illustrates how persistent fears about a global virus can generate detrimental outcomes for employees at night. An ability to fall asleep without problem or sleep through the night without interruption requires a peaceful mind, which employees upset by a crisis do not possess (Ahorsu et al., 2020; Ren, Gao, & Chen, 2020). Their depleted positive resources can be manifest as both concerns that they may not be able to provide for the economic needs of themselves or loved ones (Hayes, VanBrackle, & Sigman-Grant, 2016) and a general sense of hopelessness and anxiety (Slade, Grove, & Burgess, 2011). As mentioned at the outset, these mediating roles of economic concerns and psychological distress indicate that employees who cannot keep the threats of the pandemic out of their minds may suffer a *dual* harm: They already feel overwhelmed by the ways that the global virus has negatively affected their lives, *and then* the associated economic and psychological hardships prevent them from enjoying a good night of sleep, which could lead to even more problems (Kucharczyk, Morgan, & Hall, 2012; Scott & Judge, 2006).

Another pertinent theoretical insight is that this negative cycle can be *disrupted* by employees' attention–awareness mindfulness (Hülsheger et al., 2013). In COR theory, the harms that come with resource-draining life circumstances can be mitigated to the extent that employees counter them with valuable personal resources (Hobfoll, 2001). The chances that employees feel psychologically distressed and unable to sleep well diminish when they are more aware of and attentive to their present reality (Brown & Ryan, 2003). This resource may help employees accept and adapt to the pandemic, find meaning in it, and thus feel less distressed by it (Haun, Nübold, & Bauer, 2018; Weintraub, Pattusamy, & Dust, 2019). Mindful employees also may suffer less from their pandemic fears if they use the associated residual energy to engage with others and share their hardships (Gunasekara & Zheng, 2019), which reduces their experienced strain and associated insomnia. This study thus provides insights into how organizations can avoid the dual harm of pandemic fears and sleepless nights among their employee bases, by stimulating and honing their mindfulness.

### Limitations and future research

This study has some limitations, which set the stage for additional investigations. First, the possibility of reverse causality cannot be ignored: Employees who suffer from a persistent lack of sleep may be more sensitive to challenging life situations, such that it becomes more difficult to keep negative thoughts about a pandemic out of their minds. Our conceptual arguments are anchored in well-established COR and terror management theories, according to which the experience of resource-draining, life-threatening situations can spill over ‘into the night’ (Burke, Martens, & Faucher, 2010; Toker, Laurence, & Fried, 2015). Moreover, the time frames that we asked the respondents to reflect on stemmed from a sequential logic – the period since the start of the pandemic for fear of COVID-19, the past month for economic concerns and psychological distress, and the past few days for insomnia – which alleviates concerns about reverse causality to some extent. Yet these time frames admittedly overlap.^6^ Continued research could assess each of the focal constructs at different points in time, to estimate cross-lagged effects and formally establish causality. A related limitation is that we theorized about the resource-depleting effect of employees' fear of COVID-19, but we did not specifically assess the levels of or changes in employees' resource levels. Further longitudinal studies could address this gap.

Second, the two-study design allowed us to apply two measures of fear of COVID-19 – one that speaks to employees' preoccupations with the virus and its consequences, adapted from a fear of terror scale (Haq, De Clercq, & Azeem, 2019), and another that taps their fear in a more direct manner (Ahorsu et al., 2020) – through two *independent* data collection efforts. The consistent support for our hypotheses indicates that both measures help explain variation in employees' suffering from insomnia. Yet it would be useful if continued research were to include both measures in a single study, to assess the extent to which they overlap empirically and compare their *relative* influences on employees' insomnia. The two studies reported herein also compliment each other by pinpointing the detrimental roles of two types of hardship: economic and psychological. Further research might combine these two aspects in a single study, to assess the possible presence of *sequential* mediation (e.g., economic concerns influence psychological distress). Such research also could include a test of the buffering role of mindfulness to determine if our study 2 finding, regarding its effect on the connection between employees' fear of COVID-19 and psychological distress, applies to predictions about their worries about their economic situation too. Our focus on mindfulness also might be extended with examinations of other personal buffers that might help employees cope with their pandemic fears, such as psychological capital (Madrid, Diaz, Leka, Leiva, & Barros, 2018) or passion for work (Klaukien, Shepherd, & Patzelt, 2013).

Third, an empirical weakness of our research is that the samples in both studies included a relatively high number of female and young respondents; it would be useful to seek more diverse samples. A related limitation is our focus on one country, Pakistan. Our theoretical arguments are not country-specific, so we expect that the hypothesized relationships apply to a wide set of countries. Yet even if the nature of the relationships is unlikely to vary across countries, their *strength* might. In this regard, Pakistan represents a relevant context. The intensity with which its society has been hit by COVID-19, its precarious economic situation before and during the pandemic, and its high level of uncertainty avoidance (Hofstede, Hofstede, & Minkov, 2010) make it likely that employees experience the pandemic as highly intrusive for the quality of their daily lives (Khan, Khan, Maqsood, Hussain, Huda, & Zeeshan, 2020; Meo, Sabir, Chaudhry, Batool, & Farooq, 2020). Research thus should explicitly examine the roles of pertinent macro-level economic and cultural factors in terms of how employees respond to pandemic fears in *cross-country* studies.

### Practical implications

This study offers valuable insights for practice. Organizations must acknowledge that the negative consequences of a pandemic, such as COVID-19, cannot be underestimated. Among all its other damages, it has detrimental consequences for employees' sleep quality, as informed by their worries about unmet economic goals and their diminished psychological well-being. Insomnia is a challenge that hits many employees (Barnes, Miller, & Bostock, 2017; Crain, Brossoit, & Fisher, 2018), and a global pandemic reinforces this phenomenon. An additional challenge for organizations is that some employees may be reluctant to express their fears about COVID-19 or complain about their sleep quality, so as not to look vulnerable or weak (Ren, Gao, & Chen, 2020). Organizational decision makers accordingly are advised to create pertinent communication channels – by virtual means or in person, when the pandemic crisis allows it – through which employees can express their worries about the virus, in terms of its detrimental effects on not just their professional careers but also their private lives (Sanders, Nguyen, Bouckenooghe, Rafferty, & Schwarz, 2020). Such communication can serve a cathartic function for employees, such that they might feel relieved and find renewed energy to tackle the upsetting situation, with positive consequences for both their sleep and their work quality.

Yet the modern reality implies that preventing fears of COVID-19 and its consequences is virtually impossible. Therefore, organizations should seek to diminish or disrupt the resulting counterproductive spiral, whereby fears of COVID-19 are associated with enhanced insomnia, which may generate more preoccupations with the consequences of the virus. Employees' mindfulness is a critical personal resource in this regard (Jimenez, Niles, & Park, 2010). To the extent that organizations can make employees more mindful, and hence better able to deal with threats in their present reality, they can diminish the risk that their employee bases suffer from significant sleep problems in response to the pandemic. Organizations accordingly might benefit from evaluating and predicting employees' inclinations to be more attentive to their current reality and the corresponding probability that they avoid automatically negative responses to pandemic fears (Brown & Ryan, 2003; Haun, Nübold, & Bauer, 2018). They also could assess employees' propensities to adapt to and derive meaning from challenging work situations, which may generalize to how they handle significant challenges, inside and outside the workplace.

Finally, prior research emphasizes the value of mindfulness *intervention* programs, which could have instrumental roles in helping employees find valuable solutions to pandemic fears (Chiesa & Serretti, 2009; Hülsheger, Feinholdt, & Nübold, 2015). To enhance employees' mindfulness and its productive application, organizations could outline which individual capabilities are most needed to cope with life-threatening events, then train employees how to *leverage* those capabilities into concrete plans of action that mitigate the influences of negative events on their personal well-being. This approach may help employees increase the quality of their own lives but also enable them to contribute effectively to the collective well-being of their employing organization. A particularly useful outcome of mindfulness intervention programs, according to a comprehensive overview of them (Jamieson & Tuckey, 2017), is employee safety. Employee participation in such programs thus could diminish the chances that safety-related fears about the coronavirus enter the workplace, with potentially positive consequences for employee well-being outside work too.

## Conclusion

This research provides pertinent insights into how employees may suffer in the presence of life-threatening events, such as the COVID-19 pandemic. In particular, we detail how both economic and psychological hardships underpin the link between fears of COVID-19 and insomnia, as well as the beneficial, buffering role that employees' attention–awareness mindfulness can play in this process. Their worries about their economic situation and their psychological distress help explain how agitation about the coronavirus might be linked to persistent sleep deprivation. We also show that this detrimental process can be subdued among employees who can rely on their mindfulness, a personal resource that enables them to maintain their sleep quality despite persistent pandemic fears. Research into the harmful effects of COVID-19 is just beginning; we hope to have advanced it by detailing how organizations can contain the harms by stimulating and embracing employees' valuable, energy-enhancing personal resources.

## Footnotes

1 One item (‘I am not able to afford to buy my children the things that they need’) was removed from the analysis because of its low reliability and a factor loading below the benchmark value of .30 (Lattin, Carroll, & Green, 2003).

2 One item (‘I wake up earlier than I used to do’) was removed from the analysis because of its low reliability and low factor loading (Lattin, Carroll, & Green, 2003).

3 Even if the retained factor loadings of the measurement items varied in size (ranging between .47 and 85), they all exceeded .30 – a common cut-off value for measurement items (e.g., Deeter-Schmelz & Ramsey, 2010; Raja, Johns, & Ntalianis, 2004; Tetrick, Thacker, & Fields, 1999). Prior studies also indicate that lower fit indices are more acceptable in understudied research settings (Kashif, Braganca, Awang, & De Run, 2017; Lattin, Carroll, & Green, 2003). Research conducted in the specific country setting of Pakistan reports similar CFI, IFI and TLI values, lower than .90 but higher than .80, as acceptable (e.g., De Clercq, Azeem, & Haq, 2021; De Clercq, Jahanzeb, & Fatima, 2020c).

4 The factor loadings of the measurement items ranged between .40 and 80, and the fit indices were similar to and somewhat better than those reported in study 1. Thus, the same arguments apply with respect to their acceptability in a Pakistani context, as explained in footnote 3.

5 A post hoc analysis confirmed that mindfulness did not significantly moderate the second relationship.

6 To assess empirically whether reverse causality might be a concern, we applied the process macro to estimate a model that included a moderated mediation link between insomnia and fear of COVID-19, through psychological distress, which is the reverse equivalent of the comprehensive model tested in study 2. The index of moderation for this alternative model equaled −.016 (cf. −.033, as reported in Table 5), and the CI of this index *did* include 0 ([−.040; .006]), indicating the absence of moderation mediation. These results diminish concerns about reverse causality, though they cannot rule it out completely.
